# Healthcare providers’ views of factors influencing family planning data quality in Tshwane District, South Africa

**DOI:** 10.4102/phcfm.v14i1.3545

**Published:** 2022-11-02

**Authors:** Sophy M. Moloko, Mokholelana M. Ramukumba

**Affiliations:** 1 Department of Nursing Science, School of Health Care Sciences, Sefako Makgatho Health Sciences University, Pretoria, South Africa; 2 Department of Health Studies, Faculty of Human Sciences, University of South Africa, Pretoria, South Africa

**Keywords:** data quality, district health information system, family planning, healthcare providers, routine health information system

## Abstract

**Background:**

The family planning service requires a routine health information system (RHIS) that can produce quality data that will be used for making decisions. However, the quality of data generated is not always of a good standard. Its usefulness in making data-driven decisions in family planning service is questionable.

**Aim:**

The study intended to assess the quality of family planning data and determine healthcare providers’ (HCPs’) views on factors influencing data quality in Tshwane district.

**Setting:**

The study was carried out in 13 healthcare institutions in Tshwane district, Gauteng province, South Africa.

**Methods:**

This paper reports on the quantitative strand of a mixed methods study. A sample of 111 HCPs was selected through a stratified random sampling technique, and six months of monthly reports from 13 institutions were reviewed for data quality. A self-administered questionnaire and a tick sheet were utilised to collect data from the HCPs and to review monthly reports for data quality, respectively. The Statistical Package for the Social Sciences (SPSS) programme for Windows version 24.0.0 was used to analyse the data.

**Results:**

Data quality, mainly accuracy and timeliness, was a challenge. Many HCPs were not trained in the RHIS. They viewed several issues relating to organisational, behavioural and technical factors as barriers to data quality.

**Conclusion:**

The low quality of family planning data has a negative impact on policy development and decision-making.

**Contribution:**

There is a need for capacity building through training and supportive supervision, provision of adequate human and technical resources to enhance data quality and use of information for decision-making.

## Introduction

Family planning services can reduce maternal and infant mortality rates caused by unintended pregnancies. This would require good access to and integration of family planning services into national strategies and programmes by 2030.^1^ Quality and reliable family planning data are crucial in assessing the access, performance, quality and coverage of the family planning service. Such data are critical to understand progress towards achieving the third sustainable development goal (SDG), which is aimed at promoting wellness and health among all people.^2^ Monitoring the achievement of the SDG target relies on an efficient and effective health information system (HIS) that produces good quality data to support planning, management of services and policy development.^3,4^

Family planning data are managed through the routine HIS (RHIS), a subsystem of the HIS. The RHIS is a system designed for the routine collection, processing, use and dissemination of data to improve the management of health programmes, resources and healthcare outcomes.^4^ The system generates information at regular periods to satisfy the information requirements.^5^ Moreover, the RHIS ensures the generation, analysis, dissemination and use of timely, accurate and reliable information.^6^ The system also produces data on health statistics and indicators to evaluate progress towards universal health services coverage and to inform planning and performance monitoring.^7,8^

The RHIS in developing countries, including South Africa, uses the district HIS (DHIS) software to collect, store, report, analyse and present routine data.^8,9,10,11^ The use of DHIS to manage data in South Africa is guided by the district health management information system (DHMIS) policy and the DHMIS standard operating procedure (SOP).^10,12^ The policy is important for managing data effectively, because it outlines priorities and provides a framework for all stakeholders to operate within and direct the process of generating quality data.^8,13^ Quality data assist with the effective management of resources, which are required for the operation of the health system.^14^ Moreover, good quality data are a requirement for the delivery of safe and reliable healthcare.^15,16^

In South Africa, routine family planning data are managed at the healthcare institutions. Healthcare providers (HCPs) collect data in the form of data elements derived from national indicator data sets (NIDS) and provincial indicator data sets, which were developed by the national and provincial departments of health, respectively.^8,11^ Daily or weekly, the data are validated and captured in an electronic instrument.^12,17^ On a monthly basis, reports are compiled and sent to the district health information management (HIM) unit.^8,10,18,19^ The data are then entered into the DHIS programme for analysis.^12^

Nonetheless, the data management process is not always conducted as required, thus affecting data quality. It has been discovered that poor data quality is the primary contributor to unreliable information.^20,21,22^ Therefore, the information remains in reports, databases and on shelves without being utilised for policy development, programme enhancement or strategic planning.^23,24^

For the data to be of good quality, it is necessary to satisfy the criteria for data quality. The criteria include accuracy, relevancy, understandability, completeness, consistency, representation, reputation, security, objectivity, timeliness, precision, reliability, integrity, availability and confidentiality.^8,16,25,26^ Even though there are many data quality criteria, the literature identifies precision, completeness, timeliness and accessibility as significant data quality dimensions.^20,27^ Available, complete, timely and accurate information is essential for making decisions on formulation of policies, planning and monitoring.^27^

Several elements influencing data quality have been identified in other health programmes, including an expanded programme on immunisation and human immunodeficiency virus (HIV). The elements are categorised into organisational, behavioural and technical factors.^28^ Organisational factors include governance, training, monitoring, the availability of resources and the promotion of a culture of information use.^8,27,29,30^ Individual characteristics influencing the execution of RHIS tasks, such as knowledge and abilities, confidence, motivation and attitude, are known as behavioural factors. Finally, technical factors relate to the processes and information technologies which are used to create and manage the RHIS.^24,27,31^ The development of indicators, the design of the system and the data gathering tools are considered to be the most crucial components of the system’s technical management, which are the creation of indicators, system design, registers and data gathering tools.^24,27,30^

Considering the impact of data quality on monitoring the performance of family planning services, planning and decision-making and policy improvement, it is essential to assess the quality of family planning data, as well as the behaviour of data collectors and managers, the instruments which are used to collect data and the environment in which data are managed. Hence, the researchers sought to assess the quality of data and determine the factors influencing the quality of family planning data at Tshwane district healthcare institutions.

## Methods

### Study setting

The study was carried out in 13 healthcare institutions based on Region 3 of Tshwane district, Gauteng province. The 13 institutions included 11 primary healthcare (PHC) clinics, one community healthcare centre and one district hospital. The healthcare institutions of Tshwane district, Region 3, are in the Pretoria central business district and the surrounding suburbs, where the majority of the youth and adult population resides because of the employment opportunities. The institutions have substantial headcounts of population aged 15–49 years who are seeking family planning services; therefore, they generate significant family planning data.^32^

### Study design

The study is a quantitative phase of the explanatory sequential mixed study which was conducted to evaluate the performance of RHIS for reproductive health in Tshwane District.^8^ The quantitative approach, using a descriptive design, was applied to assess the quality of family planning data and to determine the organisational, behavioural and technical factors influencing family planning data quality.

### Study population

The study population comprised 157 HCPs working at the healthcare institutions of Tshwane district, Region 3. The population included 18 enrolled nurses, 130 registered nurses and nine medical officers or doctors.

### Study sample

The researchers selected all-region three public healthcare institutions under the management of the Tshwane district, of which there were 15; however, only 13 participated in the study. The two remaining institutions did not grant the researchers’ permission to conduct the study. Stratified probability sampling was used to select the HCPs. Based on the Raosoft online sample calculator (Raosoft Inc., Seattle, Washington, United States), with 95% confidence level, 5% margin of error and 50% response distribution, a sample size of 111 HCPs was considered sufficient to represent the population. The required sample size per stratum was calculated using the stratified sampling formula. Six doctors, 92 registered nurses and 13 enrolled nurses were considered representative for each stratum. The researcher increased the sample by 10% to 122% to increase the buffer for nonresponse. Therefore, seven doctors, 101 registered nurses and 14 enrolled nurses were selected. To select the HCPs, the researchers utilised the attendance register for all HCPs in the institutions for developing the sample frame. Subsequently, simple probability sampling was conducted using a random table to select the required sample size for doctors and registered and enrolled nurses.

### Data collection process

Data were collected from the healthcare practitioners, and monthly reports were reviewed for data quality. The researchers arranged a meeting with the facility managers for the recruitment of HCPs and data collection. The meeting took place in the morning at 07:30. The HCPs were invited to the meeting to explain the study, and those who agreed to take part in the study were selected. The researcher obtained informed consent from the selected HCPs, and the questionnaires were administered. The HCPs were given an opportunity to complete the questionnaire during their tea breaks or at lunchtime. The questionnaires were collected in the afternoon on the same day, they were distributed. A total of 122 questionnaires were distributed, and 118 were returned, equating to a 98% response rate.

The review of monthly reports for data quality using the data collection sheet took place on the same day while awaiting the questionnaires. The operational managers retrieved the monthly reports for review. The DHIS family planning software-generated reports for each institution were subsequently reviewed at the district office, assisted by the health information officer (HIO). Data were collected from March 2018 to June 2018.

### Data collection tools

The questionnaire and the data collection sheet were adapted from the US Agency for International Development (USAID) and Monitoring and Evaluation to Assess and Use Results (MEASURE) Evaluation Performance of Routine System Management (PRISM) tools, after permission was sought from the MEASURE Evaluation.^8,33^

The questionnaire was anonymous and consisted of four sections: the demographic section, followed by organisational, behavioural and technical factors. Assessment of organisational factors focused on the views of the HCPs concerning the resources available for data collection, support and supervision from HIOs and training on RHIS. Behavioural factors focused on HCPs’ understanding of the recording of family planning data elements and their perceived confidence in family planning data management tasks.^8^ The perceived level of confidence was measured on a scale from 0 to 10. Zero indicates no confidence, while 10 indicates the utmost confidence. The technical factor was the HCPs’ views concerning the effectiveness of the data collection tool in collecting family planning data. The internal consistency of the data collection instrument was established through a Cronbach’s alpha coefficient test. The tool’s reliability score was 0.875%, which exceeded the minimum acceptable score of 0.7.^8,34^

The tick sheet was used to assess the quality of family planning data generated from June to November 2017 for the availability, completeness, accuracy and timeliness of monthly reports. Data availability was assessed by reviewing the availability of RHIS reports containing family planning data. Data completeness was assessed by identifying how many contraceptive data items in the monthly reports were left blank without indicating ‘0’ when the service was not offered. The accuracy of data was assessed by comparing monthly reports generated by the institutions with those generated by the DHIS software at the district office for the same period. The following family planning data elements were assessed: oral pill cycle issued, norethisterone enanthate injection administered, medroxyprogesterone injection administered, intrauterine device (IUD) inserted and subdermal implant inserted. Observing the dates that monthly reports were sent to the subdistrict or district HIM office was used to assess the timeliness of the data. E-mails containing monthly reports were used as source evidence.^8^

### Data analysis

Data obtained from the responses were captured into a Microsoft Excel spreadsheet (Microsoft Corporation, Redmond, Washington, United States) and cleaned. Seven out of 118 questionnaires were not eligible for analysis due to missing data. Therefore, a total of 111 questionnaires were analysed using the Statistical Package for the Social Sciences (SPSS) software version 24.0.0 (IBM Corporation, Armonk, New York, United States) to obtain descriptive statistics, including the cross-tabulation by analysing the relationship between the training and the understanding of reproductive data management. The results of the data analysis were presented using tables.

### Ethical considerations

Ethical approval was granted from the Health Studies Research and Ethics Committee of the University of South Africa (UNISA), reference number HSHDC/719/2017. The study was also approved by the Tshwane Research Committee (project number 99/2017) and was registered on the National Health Research Database with the reference number GP_201711_004. Informed consent was obtained, and principles of the right to self-determination, privacy, confidentiality, anonymity and protection of harm were adhered to throughout the study.

## Results

### Findings from data quality assessment

#### Availability of monthly reports

All 13 (100.0%) institutions had electronic copies of RHIS monthly reports over the 6-month period (June 2017 – November 2017) of the review.

#### Data completeness

Monthly reports from June 2017 to November 2017 were completed in all 13 institutions (100.0%). No family planning data elements were left blank.

#### Data accuracy

In June, the recorded data elements on family planning services, mainly the administration of norethisterone enanthate injection, insertion of an IUD and subdermal implant, were accurate in all (100.0%) institutions, while he oral pill cycle issued and administration of medroxyprogesterone injection were accurate in 92% of institutions. Only one institution (3%) had inaccurate data for oral pill cycles issued in July and August 2017, while all other elements were accurate. For the oral pill cycle issued, norethisterone enanthate injection administered, medroxyprogesterone injection administered and subdermal implant inserted, the data were accurate in 38%, 31%, 31% and 62% of institutions, respectively, in September. Eighty-five percent of institutions had accurate data for the oral pill cycle issued and IUD inserted for October 2017, whereas 97% of institutions had accurate data for norethisterone enanthate and medroxyprogesterone injection administered and subdermal implants inserted. In November 2017, all five data elements were accurate across all institutions (100.0%).

#### Data timeliness

Fifty-three point eight percent of institutions submitted their reports on time to the subdistrict and district in June 2017 and July of 2017. Only 46.1% of institutions submitted their reports on time in August. In September and October, 92.3% and 84.6% of institutions submitted reports on time, signalling a more noticeable improvement. On the other hand, data timeliness decreased to 38.4% in November. In this study, 61.5% of monthly reports were delivered on time on average.

### Factors influencing data quality

#### Healthcare providers’ sociodemographic information

Table 1 shows that 83.8% of HCPs were female. The age group of the HCPs ranged from 20 to 69, with the majority (55.8%) aged between 20 and 39 years. In terms of the highest academic qualifications, 59.5% of HCPs held a diploma in nursing and 27.9% had a bachelor’s degree. The most significant proportion (82.9%) of HCPs were registered nurses, while the largest percentage (30.6%) were employed in the institution for 10–14 years.

**TABLE 1:** Healthcare providers’ demographic profiles.

Variables	Count (*n*)	Proportion (%)
**Gender**		
Male	18	16.2
Female	93	83.8
**Age group**		
20–39 years	62	55.8
40–59 years	46	44.1
60–69 years	3	2.7
**Highest academic level**		
Master’s degree	1	0.9
Bachelor’s degree	31	27.9
Nursing diploma	66	59.5
Enrolled nursing certificate	13	11.7
**Position at the facility**		
Medical doctor	6	5.4
Registered nurse	92	82.9
Enrolled nurse	13	11.7
Other	0	0.0
**Years of employment in the institution**		
0–4 years	26	23.4
5–9 years	23	20.7
10–14 years	34	30.6
15–20 years	25	22.5
> 20 years	3	2.7

*Source:* Adapted form Moloko SM. Evaluating performance of routine health information system for reproductive health in Tshwane [homepage on the Internet]. University of South Africa; 2021 [cited 2022 July 10]. Available from: https://uir.unisa.ac.za/bitstream/handle/10500/27934/thesis_moloko_sm.pdf?sequence=1&isAllowed=y

### Organisational factors

#### Healthcare providers’ views regarding the availability of resources

More than 60.0% of the HCPs agreed the DHMIS SOP, DHMIS policy and updated operational plan with family planning service plans and targets, and the latest NIDS definitions are always available when needed for reference. Concerning the adequacy of resources required for data management, few HCPs agreed that the HCPs (27.0%), data capturers (32.4%) and computers (38.7%) were sufficient for data management. However, the majority (83.8%) of HCPs agreed that the PHC data collection tool was always available (see Table 2).

**TABLE 2:** Healthcare providers’ views regarding the availability of resources.

Variables	Count (*n*)	Proportion (%)
**DHMIS SOP**		
Disagree	18	16.2
Neutral	22	19.8
Agree	71	64.0
**DHMIS policy**		
Disagree	19	17.0
Neutral	25	22.5
Agree	67	60.4
**Up-to-date operational plan indicating family planning plans and targets**		
Disagree	12	10.8
Neutral	30	27.0
Agree	69	62.2
**Current national indicator data sets definitions**		
Disagree	11	9.9
Neutral	28	25.2
Agree	72	64.9
**Adequate HCPs for data collection**		
Disagree	42	37.8
Neutral	39	35.1
Agree	30	27.0
**Sufficient data capturers for data capturing**		
Disagree	34	30.6
Neutral	41	36.9
Agree	36	32.4
**Sufficient computers for capturing data**		
Disagree	28	25.2
Neutral	40	36.0
Agree	43	38.7
**The PHC data collection tool is always available for data collection**		
Disagree	4	3.6
Neutral	14	12.6
Agree	93	83.8

*Source:* Adapted form Moloko SM. Evaluating performance of routine health information system for reproductive health in Tshwane [homepage on the Internet]. University of South Africa; 2021 [cited 2022 July 10]. Available from: https://uir.unisa.ac.za/bitstream/handle/10500/27934/thesis_moloko_sm.pdf?sequence=1&isAllowed=y

DHMIS, district health management information system; SOP, standard operating procedure; HCPs, healthcare providers; PHC, primary healthcare.

#### Views regarding support and supervision from health information officers

Table 3 shows that only 35.1%, 28.8% and 24.3% agreed that the HIO conducts supervisory facility visits at least once per quarter, checks family planning data quality during the visits and discusses family planning service performance based on the RHIS data during facility visits, respectively. A smaller proportion of HCPs agreed that HIO officers allow them to discuss health information challenges during the visit (21.6%), HIO conducts on-site training when necessary during the visit (18.9%) and HIO sent the report or feedback on the last supervisory visit (18.9%).

**TABLE 3:** Healthcare providers’ views regarding support and supervision from health information officers.

Variables	Count (*n*)	Proportion (%)
**HIO conducts supervisory visits at least once per quarter**		
Disagree	34	30.6
Neutral	38	34.2
Agree	39	35.1
**HIO checks family planning data quality during the visit**		
Disagree	37	33.3
Neutral	42	37.8
Agree	32	28.8
**During the visit, HIO discusses the performance of the family planning service based on data**		
Disagree	40	36.0
Neutral	44	39.6
Agree	27	24.3
**During the visit, HIO will allow you to discuss health information management challenges**		
Disagree	48	43.2
Neutral	39	35.1
Agree	24	21.6
**HIO conducts necessary on-site teaching or training during the visit**		
Disagree	51	45.9
Neutral	39	35.1
Agree	21	18.9
**HIO provides feedback or a report on the most recent visit**		
Disagree	43	38.7
Neutral	47	42.3
Agree	21	18.9

*Source:* Adapted form Moloko SM. Evaluating performance of routine health information system for reproductive health in Tshwane [homepage on the Internet]. University of South Africa; 2021 [cited 2022 July 10]. Available from: https://uir.unisa.ac.za/bitstream/handle/10500/27934/thesis_moloko_sm.pdf?sequence=1&isAllowed=y

HIO, health information officer.

#### Training on routine data management

**FIGURE 1:**
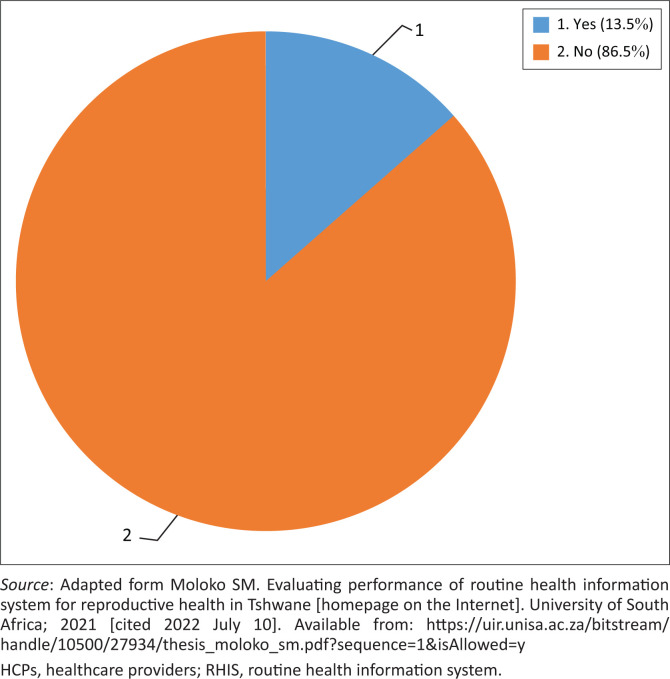
Healthcare providers trained on routine health information system.

Figure 1 shows that 86.5% of HCPs did not attend a 3–5-day training on RHIS.

### Behavioural factors

#### Understanding the recording of family planning data elements

The understanding was measured by requesting the HCPs to select the age groups of which they record a specific data element when providing the service. Approximately 30.0% of HCPs understood the recording of the administration of the norethisterone enanthate injection, medroxyprogesterone injection, oral tablet cycle, insertion of an IUD and subcutaneous implant. According to the National Department of Health (NDoH), the data elements are recorded when the service is offered to patients who are within the reproductive ages (15–49 years).^35^

The majority of HCPs understood the recording of sterilisation conducted on a man or woman (77.5%), male condoms issued (90.1%) and female condoms issued (86.5%). The data elements are recorded when the service is offered to all age groups^35^ (see Table 4).

**TABLE 4:** Healthcare providers’ understanding of the recording of family planning data elements and attending routine health information system training: cross-tabulation.

Service provided	Patients’ age groups	HCPs attended 3–5-day RHIS training				Total	
		Yes		No		*n*	%
		*n*	%	*n*	%		
Supply of oral pills	Below 15 years	0	0.0	4	4.2	4	3.6
	15–49 years	8	53.3	26	27.1	34	30.6
	All age groups	7	46.7	66	68.7	73	65.8
	Total	15	100.0	96	100.0	111	100.0
Administration of medroxyprogesterone injection	Below 15 years	0	0.0	2	2.1	2	1.8
	15–49 years	9	60.0	23	24.0	32	28.8
	All age groups	6	40.0	71	73.9	77	69.4
	Total	15	100.0	96	100.0	111	100.0
Administration of norethisterone enanthate injection	Below 15 years	1	6.6	2	2.1	3	2.7
	15–49 years	7	46.7	23	24.0	30	27.0
	All age groups	7	46.7	71	73.9	78	70.3
	Total	1	100.0	96	100.0	111	100.0
Insertion of a subdermal implant	Below 15 years	0	0.0	0	0.0	0	0.0
	15–49 years	8	53.3	26	27.1	34	30.6
	All age groups	7	46.7	70	72.9	77	69.4
	Total	15	100.0	96	100.0	111	100.0
Insertion of an IUD	Below 15 years	0	0.0	0	0.0	0	0.0
	15–49 years	6	40.0	29	30.2	35	31.5
	All age groups	9	60.0	67	69.8	76	68.5
	Total	15	100.0	96	100.0	111	100.0
Performing sterilisation	Below 15 years	0	0.0	0	0.0	0	0.0
	15–49 years	3	20.0	22	22.9	25	22.5
	All age groups	12	80.0	74	77.1	86	77.5
	Total	15	100.0	96	100.0	111	100.0
Supplying male condom	Below 15 years	0	0.0	0	0.0	0	0.0
	15–49 years	0	0.0	11	11.4	11	9.9
	All age groups	15	100.0	85	88.6	100	90.1
	Total	15	100.0	96	100.0	111	100.0
Supplying female condom	Below 15 years	0	0.0	0	0.0	0	0.0
	15–49 years	2	13.3	13	13.6	15	13.5
	All age groups	13	86.7	83	86.4	96	86.5
	Total	15	100.0	96	100.0	111	100.0

*Source:* Adapted form Moloko SM. Evaluating performance of routine health information system for reproductive health in Tshwane [homepage on the Internet]. University of South Africa; 2021 [cited 2022 July 10]. Available from: https://uir.unisa.ac.za/bitstream/handle/10500/27934/thesis_moloko_sm.pdf?sequence=1&isAllowed=y

HCPs, healthcare providers; RHIS, routine health information system; IUD, intrauterine device.

#### Relationship between healthcare providers’ understanding of recording of family planning data elements and routine health information system training

Table 4 compares the understanding of recording of the data elements between HCPs who attended the training (yes) and those who did not attend the training (no). Among HCPs who attended the training, 53.3%, 60.0%, 46.7%, 53.3% and 40% understood the recording of the oral pill, administration of medroxyprogesterone and norethisterone enanthate injection and the insertion of subdermal implant and IUD, respectively. More than 65.0% of the HCPs who did not attend the training did not understand the recording of the oral pill, administration of medroxyprogesterone and norethisterone enanthate injection and the insertion of subdermal implant and IUD. The NDoH requires the recording of the mentioned data elements when the service is offered to women aged between 15 and 49 years.^35^

Eighty percent, 100.0% and 86.7% of HCPs who attended the training understood the recording of sterilisations performed, male condoms and female condoms issued, respectively, while 77.1%, 88.6% and 86.4% of those who did not attend the training also understood the recording of the same elements. The aforementioned data elements are recorded on the RHIS when the service is provided to all age groups.^35^

#### The perceived confidence of healthcare providers in performing family planning health information management tasks

**FIGURE 2:**
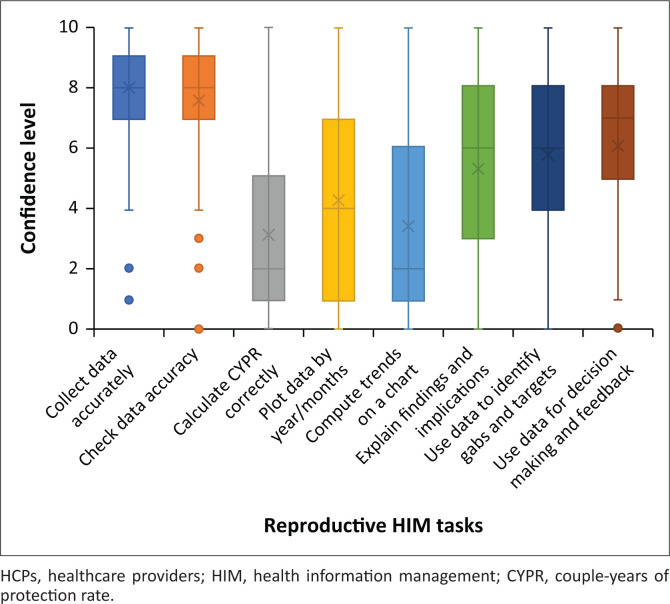
Perceived confidence of healthcare providers in performing family planning health information management tasks.

The perceived confidence of HCPs in performing family planning information management tasks was measured using a scale from 0 to 10. Zero indicates no confidence, while 10 indicates the utmost confidence. The HCPs reported that they are more confident to collect family planning data correctly (median = 8; interquartile range [IQR] = 7–9), check family planning data accuracy (median = 8; IQR = 7–9) and use data for decision-making and providing feedback (median = 7; IQR = 5–8). They are less confident in calculating the couple-years of protection rate (CYPR) correctly (median = 2; IQR = 1–5), plotting data by months or years (median = 4; IQR = 1–7) and computing trends from bar charts (median = 2; IQR = 1–6) (see Figure 2).

### Technical factors

#### Healthcare providers’ views regarding the effectiveness of the primary healthcare data collection tool in collecting family planning data

Most HCPs (68.5%) agreed that there are enough fields in the PHC tool to record information about family planning. The majority (80.2%) of HCPs agreed that it was easy to enter data into the wrong block or field on the PHC tool. Accordingly, 74.8% of HCPs agreed that it is easy to incorrectly aggregate data on a PHC tool. Some HCPs (41.4%) agreed that data collected on the PHC tool always provide an accurate representation of the family planning service activities (see Table 5).

**TABLE 5:** Healthcare providers’ views regarding the effectiveness of the primary healthcare tool in collecting family planning data.

Variables	Count (*n*)	Proportion (%)
**The PHC tool contains sufficient fields for entering family planning**		
Disagree	23	20.7
Neutral	12	10.8
Agree	76	68.5
**It is easy to enter information in the incorrect field on the PHC tool**		
Disagree	16	14.4
Neutral	6	5.4
Agree	89	80.2
**It is easy to incorrectly aggregate data on the PHC tool**		
Disagree	14	12.6
Neutral	14	12.6
Agree	83	74.8
**Data collected on the PHC tool always reflect family planning service activities accurately**		
Disagree	34	30.6
Neutral	31	27.9
Agree	46	41.4

*Source:* Adapted form Moloko SM. Evaluating performance of routine health information system for reproductive health in Tshwane [homepage on the Internet]. University of South Africa; 2021 [cited 2022 July 10]. Available from: https://uir.unisa.ac.za/bitstream/handle/10500/27934/thesis_moloko_sm.pdf?sequence=1&isAllowed=y

PHC, primary healthcare.

## Discussion

The study assessed the quality of family planning data and determined the HCPs’ views on factors influencing quality in Tshwane district.^8^ The study showed that the healthcare institutions complied with the data quality dimensions of availability and completeness. The family planning data were available and 100.0% completed in all 6-month reports assessed. The level of data completeness was high as compared to the study carried out in Ethiopia, where the service registration book and morbidity data report were 86.0% and 78.2% completed, respectively.^3^ The level of data completeness was much higher than in India, where over 80.0% of child health and reproductive reports were incomplete.^36^ The current study found variations in the accuracy of data. The lowest level of data accuracy was discovered in September 2017 on data elements measuring the provision of short-acting contraceptives, mainly on the issuing of the oral pills (38.0%), the administration of the medroxyprogesterone (31.0%) and norethisterone enanthate injections (31.0%). There were inconsistencies between data in monthly reports and the DHIS at the district office. Similarly, a low level (48.0%) of data accuracy among facility reports was reported in Ethiopia.^37^ This variation is indicative of flaws with data capturing and flow between different levels of the health system.

The data accuracy in the current study was lower than in Rwanda, where 73.3% and 70.6% of healthcare institutions transmitted data accurately from data collection tools to monthly reports and from monthly reports to the electronic database, respectively.^38^ The tallying and collation process need to be accurate, as this is the initial step in data transmission between data collection tools and a monthly report at the facility level.^39^ Inaccurate data will not truly reflect the situation as it actually exists at the institutions, which will cause a lack of confidence in the data, resulting in an inappropriate use of the information.

In this study, timely reporting seemed to be a challenge in addition to data accuracy. There were variations in how timely the monthly reports were. On average, 61.5% of monthly reports were sent to the district on time, which was slightly higher than 60.0% reported in Pakistan,^40^ much higher than 40.0% reported in Tanzania^8,20^ and, however, lower than the 75.0% recorded in Kenya.^41^ Reports that are submitted late limit the availability of data and result in incomplete portrayal of the performance of the family planning service at the district level. The time lag between data collection and submission and its analysis and dissemination to decision-makers hampers the utilisation of family planning information for decision-making.^5,8^ As a result, the data will not be useful for planning, organising and creating crucial interventions for family planning.

Regarding the organisational factors, the results showed the existence of the SOP and the policy compared to the previous study conducted in Botswana, where the documents were nonexistent.^13^ Similarly, a study conducted in South Africa found that 82.9% and 83.9% of institutions had the DHMIS SOP and DHMIS policy in place, respectively.^8,17^ The level of awareness regarding the availability of the documents was higher than in the study conducted in Kenya, where only 9% and 40% of HCPs were aware of the availability of the policy and the SOP, respectively.^8,42^ Only 16.2% and 17.0% of HCPs in this study were not aware of the availability of the SOP and the policy, respectively. The lack of awareness of the documents among HCPs in this study might reflect a lack of staff orientation on routine health information. A lack of orientation hinders HCPs’ awareness of the availability and content of the health information policy and guidelines.^42^ These documents are necessary because they govern the process of data management, including district reporting and district feedback. The HIM policy is essential for efficient data management, because it specifies priorities and offers a guiding framework for all stakeholders to function within.^8,13^

It is evident from the findings that the institutions lacked some resources for data management, mainly the HCPs and the data capturers. The finding is consistent with the previous studies, which reported a lack of personnel for managing data in Uganda and Tanzania.^43,44^ The shortage of HCPs in this study might have contributed to data collection errors, especially if the HCPs gave less attention to data management and prioritised patient care. Shortage of staff overburdens the HCPs, consequently neglecting data management and prioritising patient care.^43^ A similar study conducted in Uganda found that HCPs deemed recording of family planning data to be a secondary priority because of shortage of staff.^45^ Furthermore, a similar study in South Africa revealed that 6.9% of institutions in Gauteng province did not have data capturers.^17^ Data capturers are responsible for capturing family planning data in the electronic system to facilitate the transmission to the next level. This constraint had implications for the timeliness of reports. Work overload appeared to have contributed to data management problems, consequently compromising the RHIS’s ability to generate high-quality data.^40,43,46^

In this study, 35.1% of HCPs were supervised quarterly, which was lower than the 48.1% and 46.9% reported in Kenya and Southern Ethiopia, respectively.^47,48^ On the contrary, the proportion of HCPs supervised in this study was higher than the 31.8% and 1.3% reported in Northwest Ethiopia and Palestine, respectively.^49,50^ The frequency and the activities performed during supervision in this study seemed to be unstandardised, as shown by inconsistencies among HCPs. The inconsistent execution of supportive supervision contradicts the DHMIS SOP’s expectations. According to SOP, HIOs are intended to perform quarterly supporting supervisory visits. During the visits, they should evaluate data quality, discuss performance, discuss obstacles and offer training on-site.^12^ Inconsistent implementation of supervisory support would suggest that not all HCPs are supported. Inadequate supervisory support is also an organisational restriction that negatively impacts the utilisation of information.^24^

Training of HCPs in data management was found to be a challenge in this study, because 86.5% of HCPs did not attend the training on RHIS. This finding is consistent with reports from various regions of Ethiopia, indicating that 61.7%, 53.2% and 87.9% of HCPs lacked HIM training, respectively.^8,23,50,51^ Correspondingly, a study conducted in Kenya found that only 22.0% of HCPs were trained on HIM tasks.^42^ Inadequate training might cause employees to feel overworked and unable to fulfil their duties effectively, thus impacting data management and information utilisation.^23,52^ Moreover, it was identified as a barrier to successful data management and an indication of a lack of organisational support for data management.^8,52^

Most of the HCPs who were not trained on RHIS lacked an understanding on how to record most family planning service data, for example, the issuing of oral pills, administration of medroxyprogesterone and norethisterone enanthate injections and insertion of IUD and subdermal implant. Correspondingly, about 40.0% of HCPs who attended the training did not understand the recording of the mentioned data elements. The finding raises concerns on the quality of training provided. Training should be of good quality, covering all important aspects of data management, including the knowledge and skills needed to collect accurate data.^14^ Furthermore, the knowledge gained from training should be reinforced by the HIOs during the supportive supervisory visits.^14^ It appears that the reinforcement of knowledge did not occur in this study, as only 35.1% of HCPs received supportive supervisory visits. This might have resulted in the lack of understanding of the recording, even among the HCPs who have attended the training. Healthcare providers must record data in accordance with the NIDS definitions for the data to be correct and accurate.^8,12^ It appears that a lack of understanding of the meaning of data elements has led to inaccurate data recording and poor data quality. Similar results were found in a study conducted in Ethiopia, where over 65% of healthcare professionals lacked awareness of indicators and data management processes.^8,23^ A lack of understanding of indicators among data collectors has a negative impact on data quality and indicates the need for training and ongoing updates.^53^ Inability to understand data management prevents HCPs from generating the service delivery data required for decision-making.^53,54^

Similar to the study conducted in Kenya, the current study found a discrepancy between the actual competence in generating accurate data and self-perceived confidence among the HCPs. The majority of HCPs did not understand the recording of data elements, despite their high self-perceived confidence in the ability to gather and verify data accuracy.^8,41^

The data collection tool seemed complex, because most of HCPs revealed that it is easy to enter data into the incorrect field and aggregate it incorrectly. In a similar way, the data gathering tools in Pakistan, Nigeria and Benin were found to be complicated and contain numerous unnecessary columns.^55,56,57^ The complexity of the tool might have affected the accuracy of data as a result of incorrect data entries. In addition, the manual recording of services increases the probability of selecting the incorrect column in the data collection tool.^58^ Poorly designed and complex tools may compromise the accuracy of the data; hence, the structure of the data gathering tool should be standardised and easy.^59^

## Strength and limitations

The authors believe that the strength of this paper is the clear articulation of the interplay between organisational, behavioural and technical factors and their possible impact on data management process. The combination of the assessment of data quality and self-reported questionnaires enhanced the validity of the study. A limitation is that the study focused on the concepts in the PRISM framework and did not measure other factors that might have had an impact on the quality of data. It did not assess the degree of association between the factors and the quality of data. In addition, the study was conducted in one district, and the sample was small. However, the findings could be generalised in other districts with a similar context.

## Conclusion

The study revealed how organisational, behavioural and technical factors interact in data management. The availability of the policy and the SOP reflects good organisational governance in data management. Inadequate training, a lack of resources and insufficient supervision, which have a negative impact on staff competence and capacity for data management, were also observed to have an influence on behavioural issues. The effect of the data collection tool on data quality is highlighted. There is a need for capacity building through training and supportive supervision, as well as the provision of adequate human and technical resources, to improve data quality.

Therefore, the following recommendations were drawn from the findings of this study:

- The training managers should design and schedule frequent training sessions for HCPs that address all the necessary competencies for effective data management.
- The institutional managers should ensure that HCPs are provided with mentoring and resources to enhance their data management skills.
- The HIMs and officers are stewards of the RHIS; they should provide data quality assurance mechanisms, such as quarterly supportive supervision and data quality assessments.
- The NDoH should ensure that the data collection tool is simple to prevent errors and provide sufficient human and technical resources.
